# Comparison of Erythrocyte Membrane Lipid Profiles between NAFLD Patients with or without Hyperlipidemia

**DOI:** 10.1155/2020/9501826

**Published:** 2020-09-16

**Authors:** Wenbin Chen, Shanshan Shao, Hu Cai, Jie Han, Tian Guo, Yilin Fu, Chunxiao Yu, Meng Zhao, Tao Bo, Zhenyu Yao, Jiajun Zhao, Qunye Zhang, Guowang Xu, Chunxiu Hu, Ling Gao

**Affiliations:** ^1^Shandong Provincial Hospital Affiliated to Shandong First Medical University, Jinan, China; ^2^Shandong University, Jinan, China; ^3^Scientific Center, Shandong Provincial Hospital Affiliated to Shandong First Medical University, Jinan, China; ^4^Shandong Provincial Key Laboratory of Endocrinology and Lipid Metabolism, Jinan, China; ^5^Department of Endocrinology, Shandong Provincial Hospital Affiliated to Shandong University, Jinan, China; ^6^Key Laboratory of Cardiovascular Remodeling and Function Research Chinese Ministry of Education and Ministry of Public Health, The State and Shandong Province Joint Key Laboratory of Translational Cardiovascular Medicine, Qilu Hospital of Shandong University, Jinan, China; ^7^CAS Key Laboratory of Separation Science for Analytical Chemistry, Dalian Institute of Chemical Physics, Chinese Academy of Sciences, Jinan, China

## Abstract

Objectives. Nonalcoholic fatty liver disease (NAFLD) and hyperlipidemia (HL) are common metabolic disorders due to overnutrition and obesity. NAFLD is often associated with hyperlipidemia. The aim of this study was to identify and compare the erythrocyte membrane lipids profile in NAFLD patients with or without HL. *Methods*. A total of 112 subjects (with similar age and body mass index) were divided into four groups: (1) normal controls, (2) NAFLD alone, (3) HL alone, and (4) NAFLD combined with HL (NAFLD + HL). Lipid was extracted from the erythrocyte membrane, and lipid profiles of subjects were analyzed by liquid chromatography mass spectrometry (LC-MS). *Results*. Data sets from 103 subjects were adopted for lipidomic analysis. Significant changes of lipid species were observed in patient groups, especially in the HL group and NAFLD + HL group. The HL group showed increased level of most lipid species, and decreased level of most lipid species was observed in the NAFLD + HL group. The weight percent of myristic acid, stearic acid, erucic acid, and docosahexaenoic acid also showed distinct variation between different groups. *Conclusions*. NAFLD, HL, and NAFLD + HL all had an impact on lipid profiling of the erythrocyte membrane. The influence of NAFLD alone is less important compared with HL. Some lipids should be highlighted because of their specific role in cell function and systemic metabolism.

## 1. Introduction

Nonalcoholic fatty liver disease (NAFLD) is the nonalcohol-induced deposit of extra fat in hepatocytes, resulting in diseases ranging from hepatic steatosis (NAFL), steatohepatitis (NASH), fibrosis, and eventual cirrhosis and cancer. The prevalence of NAFLD worldwide ranges from 6.3% to 33% in the general population [1]. Importantly, people with NAFLD have an increased risk of developing type 2 diabetes and cardiovascular problems [2–4]. NAFLD's pathogenic mechanisms are still under investigation, and obesity, triglycerides (TGs) accumulation, and inflammatory response are possible pathogenic factors. Increased liver load with lipids such as free fatty acids (FFA) [5] was shown to contribute to altered hepatic metabolism. Increased serum triglycerides, cholesterol, and low-density lipoproteins (LDL), combined with decreased high-density lipoproteins (HDL), represent acceleration in cardiovascular disease incidence. The presence of hyperlipidemia (HL) was reported in 20% to 80% cases associated with NAFLD [4]. NAFLD may impair hepatic lipid metabolism and initiate hyperlipidemia [6].

As one of the most important functional components of blood, erythrocytes deliver oxygen, as well as carbon dioxide, and maintain systemic acid/base homeostasis. The state of the erythrocyte should not be neglected in exploring many disease processes [7–9]. For example, the deformation and viscosity of erythrocytes play critical roles in cardiovascular risks in patients with metabolic syndrome [10]. The structure and composition of the erythrocyte membrane represent its function in healthy or disease states [11]. The uniquely shaped lipid bilayer membrane is mainly composed of cholesterol and phospholipids. Erythrocyte deformability is related to the content of membrane phospholipids [12]. Phospholipids, as well as other lipid species, also participate in multiple pathways including metabolism and inflammation. Profiling of erythrocyte membrane lipids has been performed on several diseases [13–15] to identify the potential biomarker or pathogenic pathways. The correlations between hyperlipidemia and the erythrocyte membrane lipid composition were previously reported [12, 16]. Increased levels of lipid peroxidation products were observed in the serum and the liver of patients with NAFLD [17]. The fatty acid profile of the erythrocyte membrane from patients with NAFLD subjected to dietary intervention has been measured, and some FFAs may be selected as a potential biomarker of the disease regression [18]. Patients with NAFLD showed FA composition alterations of erythrocyte phospholipids [19].

Lipidomics was utilized to investigate the liver lipidome of NALFD [20]. However, till now, comprehensive lipids profile of the erythrocyte membrane from NAFLD and hyperglycemia patients were not investigated. A detailed research of the lipid in the erythrocyte membrane of NAFLD and hyperlipidemia patients will provide novel insights into the etiology of these diseases and choice for future biomarker selection and treatment.

In the present study, we aimed to identify and compare the erythrocyte lipids profile in four different groups of individuals with and without NAFLD or hyperlipidemia by liquid chromatography coupled with a mass spectrometer (LC-MS).

## 2. Materials and Methods

### 2.1. Study Design and Subjects

A total of 112 subjects, including normal controls and patients with nonalcoholic fatty liver disease (NAFLD) alone, hyperlipidemia (HL) alone, and NAFLD combined with HL (NAFLD + HL), were enrolled. The study population was derived from the REACTION study [21]. The subjects diagnosed within each group were with similar age and body mass index (BMI). Subjects were diagnosed with NAFLD by the quantitative ultrasound method without any other liver disease or medication taken associated with fatty infiltration of the liver [22].

Subjects with at least one of the following criteria in the serum lipid profile were recognized as hyperlipidemic: a total cholesterol (TC) level above 5.7 mmol/L, TG level above 5.7 mmol/L, HDL-C level below 0.8 mmol/L, and LDL-C level above 2.7 mmol/L [23]. The laboratory did not have any private information about the studied groups.

In this study, we excluded subjects using the following exclusion criteria to ensure that our study was not perplexed by unforeseen factors: (1) fatty liver associated with alcoholic liver disease, chronic viral hepatitis, autoimmune liver disease, hepatolenticular degeneration, congenital syndromes of severe insulin resistance, or hypo-*β*-lipoproteinemia; (2) intake of medications that influence lipid metabolism; and (3) total parenteral nutrition, inflammatory bowel disease, hypothyroidism, hyperthyroidism, or Cushing syndrome.

The experimental protocol was approved by Shandong Provincial Hospital, and all subjects provided written informed consent.

### 2.2. Blood Sampling

Fasting blood samples were collected from subjects at 8 : 00–10 : 00 a.m. after an overnight fasting. For each sample, 3 ml of blood was collected in heparinized tubes and centrifuged at 3000 rpm at 4°C for 15 min after inverting tubes gently. After centrifugation, plasma was separated for biochemical tests and frozen at −80°C for further experiments. Biochemical tests including TC, TG, LDL-C, HDL-C, and fasting blood glucose were performed in the clinical laboratory in Shandong Provincial Hospital. Erythrocytes were washed in PBS 3 times, and aliquots of 500 *μ*l were stored at −80°C for membrane lipid extraction. All samples were deidentified prior to biochemical analysis.

### 2.3. Erythrocyte Membrane Purification and Lipid Extraction

Erythrocyte cell membranes were prepared by lysis with hypotonic buffer (10 mmol/L Tris-HCl, pH 7.4), after incubation in 4 degrees for 2 hours. Cell membranes were precipitated by centrifugation at 12000 g for 15 min and washed 3 times to eliminate hemoglobin contamination.

Membrane lipids were extracted according to Folch [24]. Briefly, 750 *μ*l methanol containing lipid internal standards was added to the membrane precipitation. Then, 1 ml chloroform was added, and after incubation for 1 hour, 450 *μ*l water was added to induce phase separation. After centrifugation at 2300 g for 10 min, the lower (chloroform) phase was collected, lyophilized, and reconditioned by an injection solvent (65% acetonitrile, 30% isopropano, 5% H_2_O).

### 2.4. LC-MS Lipidomics Analysis

Erythrocyte membrane lipidomics analysis was performed by ultra-high-performance liquid chromatography (Waters, Milford, MA) coupled with a triple quadrupole time-of-flight mass spectrometer UPLC-Q-TOF/MS (AB SCIEX 5600 Q-TOF, Framingham, MA) system [25]. For separation, a C8 ACQUITY column (2.1 mm × 100 mm × 1.7 *µ*m, Waters, Milford, USA) was used. Mobile phases were as follows: A was 60% acetonitrile (acetonitrile/H_2_O = 6 : 4, 10 mM ammonium acetate) and B was isopropanol/acetonitrile = 9 : 1, 10 mM ammonium acetate; flow rate 0.26 ml/min; 32% B for 1.5 min, linear increase to 85% B in 14 min, then increase to 97% B, kept for 2.4 min, and then decrease 97% B to 32% B within 0.1 min and maintained 1.9 min. Mass spectrometry was recorded under both positive and negative electron-spray ionization (ESI) modes, voltages 4500 V for ESI− and 5500 V for ESI+, respectively. The declustering potential was 100 V, curtain gas was 35 psi, and interface heater temperature was 600°C (ESI−) and 500°C (ESI+), respectively. For MS/MS analysis, information-dependent acquisition (IDA) was applied with the collision energy of 10 V.

### 2.5. Data Processing and Statistical Analysis

Lipidview software (version 1.2, AB SCIEX, USA) was utilized to select candidate lipids. Lipid identification was based on exact mass, retention time, and MS/MS pattern. Peakview workstation (AB SCIEX, USA) was used to check MS/MS information of lipids. Multiquant (version 2.1, AB SCIEX, USA) was employed to calculate the area of lipid peaks. Internal standards, Ceramide (Cer) 17 : 0, Lysophosphatidylcholine (LPC) 19 : 0, phosphatidylcholine (PC) 38 : 0, phosphatidylethanolamine (PE) 30 : 0, SM 12 : 0, and Triglyceride (TG) 45 : 0 were used to correct lipid species in the positive mode, and LPC 19 : 0, PE 30 : 0, and free fatty acid (FFA) C16:0-d4 were used to correct lipid species in the negative mode.

Data were analyzed by Graphpad Software (San Diego, CA) and were expressed as the mean ± standard deviation (SD). Repeated measures were compared by the nne-way ANOVA test followed by a Bonferroni post hoc performance test for comparisons among four groups, and a *P* value < 0.05 was considered significant.

## 3. Results

### 3.1. Clinical Characteristics of the Subjects

Clinical parameters showed that there was no difference in the average ages, BMI, ALT, and AST among the four groups. Compared with that in the control or NAFLD group, participants from HL alone and HL + NAFLD groups displayed significantly higher serum TC, TG, and LDL-C levels and lower serum HDL-C levels. Significantly increased indirect bilirubin (IBIL) was observed in the HL alone group (Table 1).

### 3.2. Lipid Profiling of the Erythrocyte Membrane Based on LC-MS Studies

The lipid from the erythrocyte membrane of the subjects was extracted and was subjected to LC-MS analysis. Data from 9 subjects were considered as outliers and were removed from the data sets. A total of 9 lipid species and 249 lipids were identified and measured: free fatty acids (FFAs), phosphatidylcholine (PC), phosphatidylethanolamine (PE), Phosphatidylserine (PS), Phosphatidylinositol (PI), Lysophosphatidylcholine (LPC), Lysophosphatidylethanolamine (LPE), ceramide (Cer), and sphingomyelin (SM). Among these lipids, significant content and weight percent alterations were observed, especially in the HL group and NAFLD + HL group.

### 3.3. Free Fatty Acids

**Figure 1:**
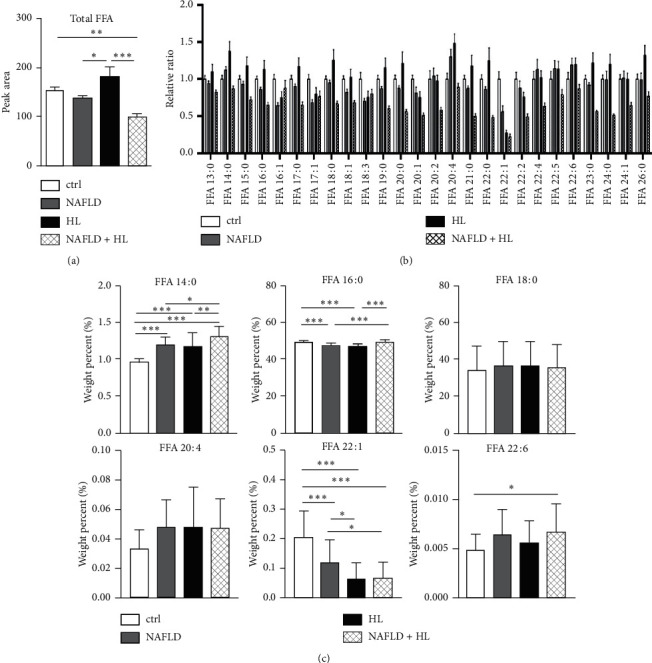
The contents of total FFA (a), relative ratio of individual FFA (b), and weight percentage (%) of six FFAs (c) in the erythrocyte membrane. Error bars represent the mean ± SD; ^*∗*^*p* < 0.05, ^*∗∗*^*p* < 0.01, ^*∗∗∗*^*p* < 0.001.

Twenty-six FFAs, including 13 saturated FFAs (SFAs, double bond = 0), 6 monounsaturated FFAs (MUFAs, double bond = 1), and 7 polyunsaturated FFAs (PUFAs, double bond ≥ 2), were measured (Figure 1(a)). The total FFA content was significantly higher in the HL group compared with that in NAFLD and NAFLD + HL groups, respectively. However, the content of total FFAs significantly decreased in the NAFLD + HL group compared with the control group (Figure 1(b)).

The relative weight percent of each FFA was calculated. Compared with the control group, there were significantly higher levels of FFA 14 : 0 (myristic acid, MA) in the 3 other groups. A marked decrease of myristic acid was observed in NAFLD + HL groups compared with NAFLD and HL group, respectively. Both NAFLD and HL groups exhibited decreased FFA 16 : 0 (palmitic acid, PA) level compared with control and NAFLD + HL groups, respectively. No difference was observed in the level of FFA 18 : 0 (stearic acid). An increase of FFA 20 : 4 (arachidonic acid) was observed in NAFLD, HL, and NAFLD + HL groups compared with control group; however, these differences did not reach statistical significance (*p*=0.07, 0.07 and 0.09, respectively). Compared with the control group, significantly decreased levels of FFA 22 : 1 (erucic acid) were shown in the 3 other groups. The NAFLD + HL group exhibited higher FFA 22 : 6 (docosahexaenoic acid) level compared with the control group (Figure 1(c)).

### 3.4. Phospholipids (PI, PC, PE, and PS)

**Figure 2:**
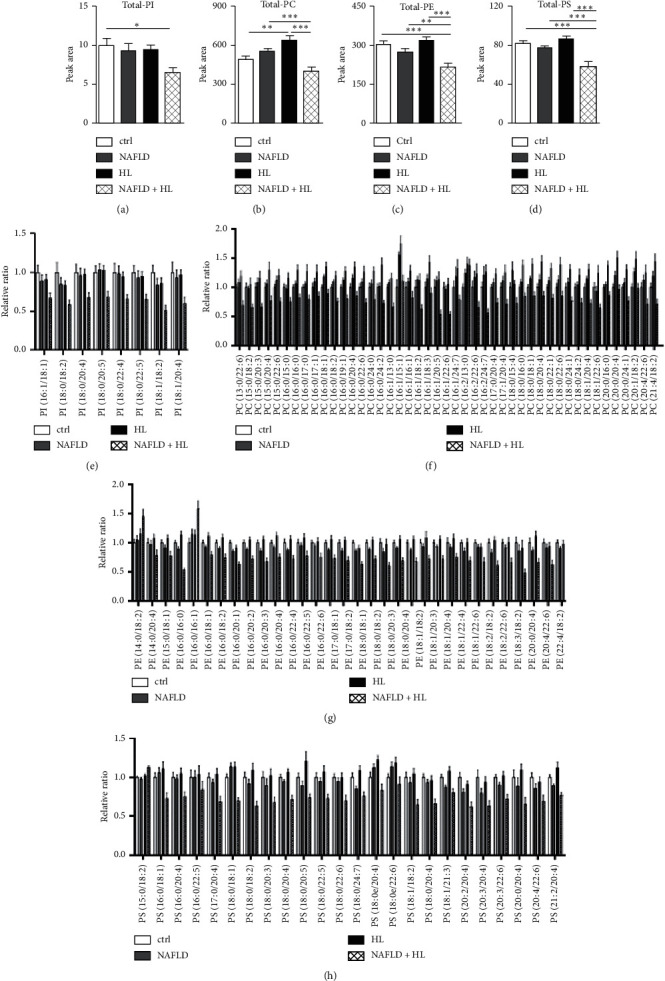
Comparison of the contents of total PI (a), PC (b), PE (c), and PS (d), relative ratio of individual PI (e), PC (f), PE (g), and PS (h) in the erythrocyte membrane between four groups. Error bars represent the mean ± SD; ^*∗∗*^*p* < 0.05, ^*∗∗*^*p* < 0.01, ^*∗∗∗*^*p* < 0.001.

Eight individual PI were detected and measured (Figure 2(e)). The NAFLD + HL group showed significantly decreased level of total PI compared with the control group. Total PI level showed no difference among control, NAFLD, and HL groups (Figure 2(a)). Thirty-two individual PE and 24 individual PS were detected and measured (Figures 2(g) and 2(h)), and the NAFLD + HL group showed significantly decreased levels of total PS, as well as total PE compared with 3 other groups (Figures 2(c) and 2(d)). No difference of total PI, PE, and PS levels among control, NAFLD, and HL groups was observed. Forty-five individual PC were detected and measured (Figure 2(f)). The NAFLD + HL group showed significantly decreased level of total PC compared with NAFLD and HL groups, respectively. Significant increase in the total PC level was observed in HL groups compared with control and NAFLD groups, respectively (Figure 2(b)).

### 3.5. Lysophospholipids

**Figure 3:**
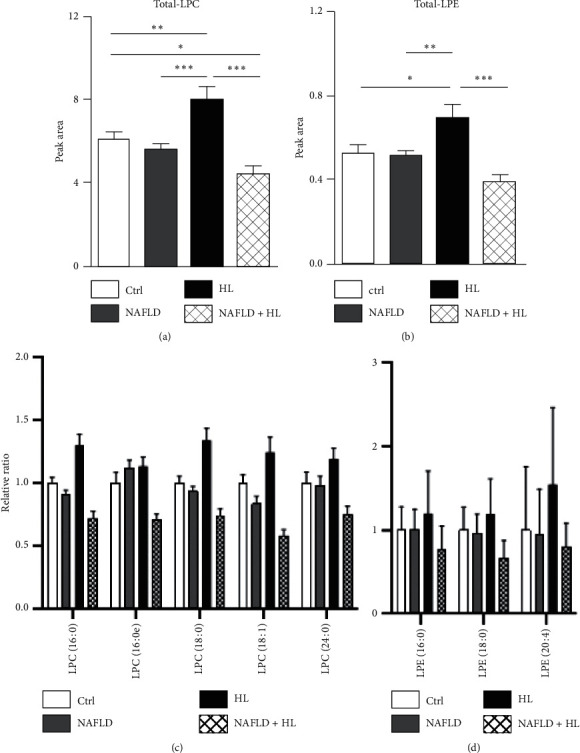
Comparison of the contents of total LPC (a), relative ratio of individual LPC (c), total LPE (b), and relative ratio of individual FFA (d) in the erythrocyte membrane between four groups. Error bars represent the mean ± SD; ^*∗*^*p* < 0.05, ^*∗∗*^*p* < 0.01, ^*∗∗∗*^*p* < 0.001.

As shown in Figures 3(c) and 3(d), 5 LPC and 3 LPE were identified and measured. The HL group showed significantly increased levels of LPC and LPE compared with 3 other groups, while the NAFLD + HL group showed notably decreased total LPC compared with the control group (Figures 3(a) and 3(b)). Neither total LPC nor LPE level showed a significant difference between the NAFLD and control group.

### 3.6. Plasmalogens (PC P and PE P) and Ether Phosphlipids (PC O and PE O)

**Figure 4:**
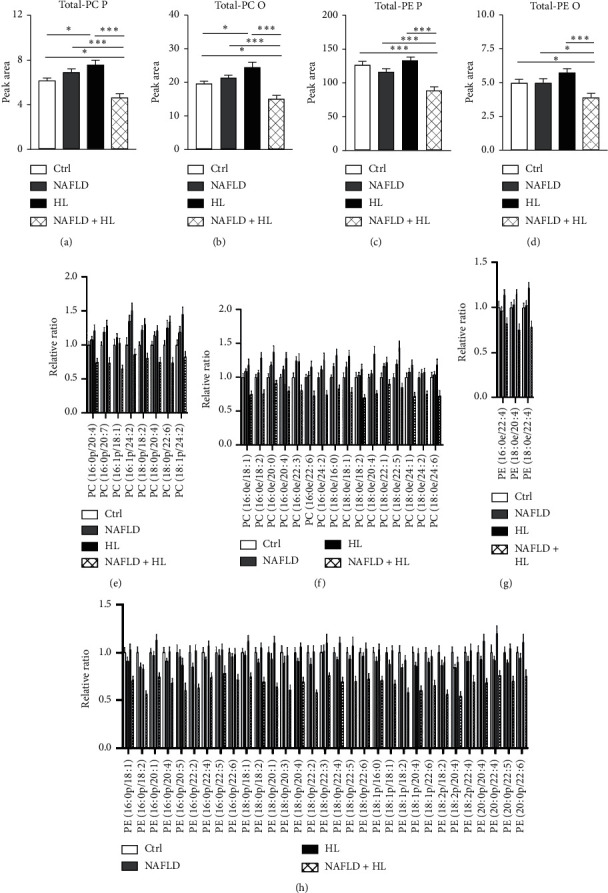
Comparison of the contents of total PC P (a), PC O (b), PE P (c), and PE O (d), relative ratio of individual PC P (e), PC O (f), PE O (g), and PE P (h) in erythrocyte membrane between four groups. Error bars represent the mean ± SD; ^*∗*^*p* < 0.05, ^*∗∗*^*p* < 0.01, ^*∗∗∗*^*p* < 0.001.

Eight PC P and 16 PC O were detected and measured (Figures 4(e) and 4(f)). The NAFLD + HL group showed marked reduction of both total PC P and PC O compared with 3 other groups, respectively. Compared with the control group, total PC P and PC O levels significantly increased in the HL group, while control and NAFLD groups are similar in terms of total PC P and PC O levels (Figures 4(a) and 4(b)). Three PE O and 31 PE P were detected and measured (Figures 4(g) and 4(h)). The NAFLD + HL group showed significant decrease of both total PE O and PE P levels compared with 3 other groups, respectively, while no difference was observed among control, NAFLD, and HL group (Figures 4(c) and 4(d)).

### 3.7. Sphingolipids (Ceramides and Sphingomyelins)

**Figure 5:**
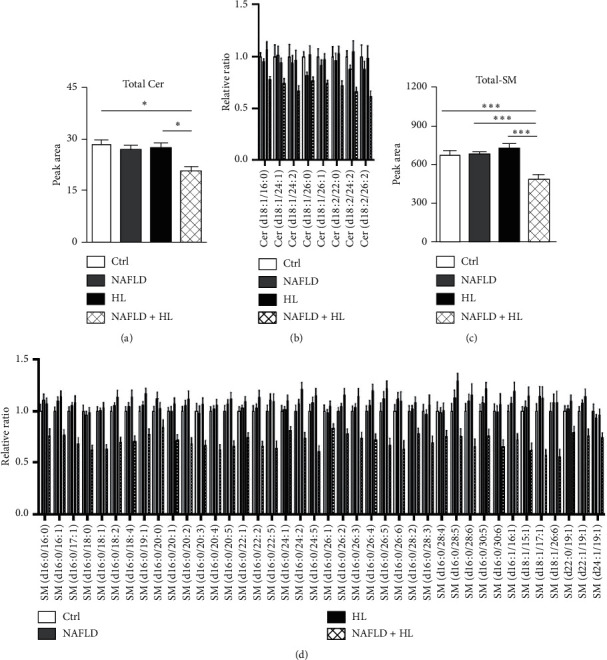
Comparison of the contents of total Cer (a), relative ratio of individual Cer (b), total SM (c), and relative ratio of individual SM (d) in the erythrocyte membrane between four groups. Error bars represent the mean ± SD; ^*∗*^*p* < 0.05, ^*∗∗*^*p* < 0.01, ^*∗∗∗*^*p* < 0.001.

Eight individual Cer (Figure 5(b)) and 40 individual SM (Figure 5(d)) were detected and measured. The NAFLD + HL group showed a marked decreased level of total ceramide compared with the control and HL group, respectively (Figure 5(a)). Compared with 3 other groups, a significant decrease in total SM was observed in the NAFLD + HL group (Figure 5(c)). Similar total levels of both Cer and SM were displayed in control, NAFLD, and HL groups.

## 4. Discussion

In this study, we investigated the alterations of erythrocyte membrane lipid profiling of grouped subjects with NAFLD, hyperlipidemia, or NAFLD accompanied with hyperlipidemia using an LC-MS based lipidomics approach. Differentially changed lipid species, as well as quite a lot of individual lipids, were found among these groups. Minor lipid profile changes were observed between the NAFLD group and control group. The erythrocyte membrane of the HL group exhibited the highest levels of most lipid species. However, most lipid species except TGs showed notably decrease in the erythrocyte membrane in the NAFLD + HL group compared with other groups. Weight percent alteration of several individual FFAs were observed among different groups. These results indicated that NAFLD, HL, and NAFLD + HL all had a close relation with the lipid profiling of the erythrocyte membrane.

Research in lipidomics provides approach to a full understanding of the whole-body metabolism status. Lipids are important constituents of cell membrane, as well as signalling molecules, and, therefore, become powerful tools to evaluate molecular unbalances of the individual [26]. The plasma lipid level reflects recent (weeks) lipid intake, while the erythrocyte membrane represents both long-term (one-two month) systemic lipid metabolism conditions because of less sensitivity to recent intake of nutrients and the refresh rate [27, 28]. The erythrocytes do not synthesize lipids due to lack of organelles. Thus, the lipid composition of the erythrocyte membrane is mainly determined by exchange with circulating lipids [29]. A long period of the maturation process (90 days) and stay in circulation (120 days) makes erythrocytes reliable cell samples to be examined. Studies have revealed the changes of plasma lipids in hyperlipidemia or NAFLD [30, 31]. Previous investigations have been performed to quantify the levels of lipids in the erythrocyte membrane in different diseases [13, 14, 32]. Our results indicated that systemic lipid metabolism disorder was associated with alterations of lipid composition of the erythrocyte membrane.

Liver steatosis is associated with hyperlipidemia, but neither every hyperlipidemia patient suffers from NAFLD nor every NAFLD patient suffers from hyperlipidemia. They suggested that the body was under the disorder status of lipid metabolism. NAFLD, HL, and NAFLD + HL patients were enrolled in this study to clarify the changes of erythrocyte membrane and their potential interactions.

Consistent with the elevated serum TG or cholesterol level, we detected increased total erythrocyte membrane FFAs and most saturated individual FFAs in the HL group. The longer- and the less-double band the FA chain, the higher the van der Waals forces and the lower the FFA fluidity, as reflected by a high FA melting point [33], and this means less fluidity of erythrocytes from HL patients. However, the NAFLD + HL group showed decreased total FFAs compared with the control and HL group, indicating that there was no additive effect as what we assumed, and excessive liver lipid deposit accompanied by hyperlipidemia did not mean surplus membrane synthetic materials for erythrocytes.

The weight percent (proportion) is often used to assess the fatty acid composition of products independently of the total fat content. The changes of several individual FFAs, here, attracted our attention. The MA proportion was significantly elevated in disease groups in comparison with control group, and studies have reported that the serum-free MA proportion increased in NASH patients [34], and MA enhanced palmitic acid-induced ER stress, as well as hepatocyte apoptosis [35]. The alteration of MA in these patients may increase the susceptibility of inflammation and injury in the liver. The decrease in the PA proportion in NAFLD and HL groups was observed. In NAFLD patients, liver fibrosis is independently associated with PA [36], and erythrocyte PA was not associated with T2D [37]. The potential effect of PA on NAFLD remains to be explored. Arachidonic acid (AA), a key inflammatory intermediate, showed the most remarkable increase in both NAFLD and HL groups. Interestingly, the relative weight percent of AA increased in all disease groups, which indicated the potential inflammation induction effect of both diseases although the increase was not statistically significant [38–40]. Erucic acid, as an omega-9 monounsaturated fatty acid, was proven to decrease the endogenous TG level and increase the TG hydrolysis rate in the heart [41], indicating its potential role in maintaining lipid hemeostasis. DHA increased in the NAFLD + HL group, but the effect of such omega-3 fatty acid on lipid metabolism and vascular events remains conflicting [42]. Another study has found that DHA prevented increased serum glucose and the ALT level in mice fed high-fat diet [43]; in this study, subjects with diabetes and abnormal liver-enzyme level were excluded, and this may partially contribute to the increased DHA weight percent in these patients.

The contents of phospholipids, Cers, and SMs all decreased in the NAFLD + HL group. Cers are proinflammatory and play important roles in suicidal erythrocyte death [44, 45]. Inhibition of ceramide synthesis was proven to prevent atherosclerosis, insulin resistance, and diabetes. Studies have found that plasma sphingomyelin levels correlate with subclinical atherosclerosis independently of cholesterol levels [46]. Deformability and survival of erythrocyte, as well as endothelial function and vascular integrity, are negatively influenced by the incorporation of lysophospholipids into the membrane [47, 48]. LPE and LPC can induce membrane microfragmentation and cause hemolysis or necrosis of erythrocytes [47, 49]. The HL group diplayed notably higher level of both LPC and LPE compared with 3 other groups, indicating that hyperlipidemia may be the most significant factor contributing to erythrocyte destruction. The increased LPC and LPE may be contributed to the elevated serum IBIL in these HL participants. The lipid bilayer of erythrocytes is mainly composed of PC, PE, PS, PI, and SM. Membrane asymmetry is maintained by these components, with PC and SM mainly located on the outer leaflet while PS and PE predominantly on the inner leaflet [50]. PE is important in increasing rigidity of the bilayer and maintaining membrane fluidity [51]; however, the PE and PC content would increase in aged erythrocytes [52]. PS translocation to the erythrocyte surface may lead to thrombosis. However, the biological function of the total PS and PI have not been well investigated and required to be explored in the future. Studies have revealed that plasmalogen-deficient cells were more sensitive to cell death [53], and PC O and PE O decreased in individuals with insulin resistance [54]. The decreased plasmalogens and ether phosphlipids in the NAFLD + HL group indicated that they are more prone to erythrocyte damage and diabetes.

In conclusion, we showed that NAFLD and HL had an impact on lipid profiling of the erythrocyte membrane. Most lipid species were increased in the HL group and decreased in the NAFLD + HL group compared with control. The lipid composition was slightly influenced by NAFLD itself, but the changes of some individual lipids drew our attention. Alterations of the lipids in NAFLD or HL patients may contribute to the increased risk for cell damage, inflammation, and metabolic abnormalities. Further studies are required to explore the effect of NAFLD or HL on membrane lipids synthesis and the metabolism pathway.

## Figures and Tables

**Table 1:** Clinical parameters in subjects from four groups.

	Ctrl	NAFLD	HL	HL + NAFLD

*n*	24	27	26	26
Gender (male/female)	10/14	11/16	11/15	12/14
Age (years)	50.75 ± 5.68	51.00 ± 5.63	50.46 ± 5.68	50.77 ± 6.05
BMI	26.20 ± 2.80	26.48 ± 2.72	25.99 ± 2.62	26.63 ± 3.00
ALT (U/L)	15.71 ± 6.05	19.67 ± 6.16	15.12 ± 6.05	19.23 ± 8.70
AST (U/L)	21.88 ± 4.17	24.00 ± 4.13	22.08 ± 4.17	25.46 ± 7.03
TBA (*μ*mol/L)	6.89 ± 4.01	5.46 ± 2.61	5.52 ± 4.01	5.77 ± 3.06
TC (*μ*mol/L)	4.59 ± 0.79	4.94 ± 1.24	5.90 ± 0.79^*∗*^^#^	5.62 ± 1.20^^*∗*^^
TG (*μ*mol/L)	0.93 ± 0.32	1.08 ± 1.34	2.05 ± 0.32^*∗*^^#^	2.32 ± 0.95^*∗*^^#^
TBIL (*μ*mol/L)	13.74 ± 2.03	14.76 ± 1.58	16.12 ± 1.67	15.26 ± 1.48
DBIL (*μ*mol/L)	5.62 ± 1.69	5.24 ± 1.10	4.82 ± 0.74	4.8 ± 1.05
IBIL (*μ*mol/L)	8.12 ± 1.30	9.52 ± 1.77	11.3 ± 1.37^*∗*^	10.46 ± 1.51
LDL-C	2.50 ± 0.60	2.93 ± 1.01	3.66 ± 0.60^*∗*^^#^	3.58 ± 0.78^*∗*^^#^
HDL-C	1.46 ± 0.30	1.28 ± 0.33	1.22 ± 0.30^*∗*^	1.19 ± 0.23^*∗*^

All values are expressed as mean ± SD;  ^*∗*^*p* < 0.05 compared with control, ^#^*p* < 0.05 compared with NAFLD.

## Data Availability

Data can be obtained from the corresponding author on request.
